# Pneumonia caused by *Schizophyllum commune* in a patient with diabetes: A case report and comprehensive literature review

**DOI:** 10.1097/MD.0000000000033773

**Published:** 2023-06-02

**Authors:** Xing Chen, Jian Sun, YeFeng Chen, Jie Wang, ShuYing Liu

**Affiliations:** a Department of Respiratory Medicine, Shaoxing People’s Hospital (Shaoxing Hospital, Zhejiang University School of Medicine), Shaoxing, Zhejiang Province, China; b Department of Respiratory Diseases, The First Affiliated Hospital of College of Medicine, Zhejiang University, Hangzhou, Zhejiang Province, China; c Department of Cardiology, Shaoxing People’s Hospital (Shaoxing Hospital, Zhejiang University School of Medicine), Zhejiang Province, China.

**Keywords:** antifungal agents, clinical presentation, pneumonia, *Schizophyllum commune*

## Abstract

*Schizophyllum commune (S. commune*) is a basidiomycete bracket fungus that rarely causes invasive fungal infections. It is often misdiagnosed as other invasive fungal disease because of its atypical clinical features. Here we report a case of pneumonia due to *S commune* and review the relevant literature.

**Patient concerns and diagnoses:**

A 55-year-old male with a history of diabetes and poor glycemic control presented with cough and sputum for half a month. Laboratory examination showed elevated peripheral blood eosinophils, bronchoalveolar lavage fluid eosinophils and increased serum total immunoglobulin E. Chest computed tomography revealed a gloved finger sign and consolidation in the middle lobe of the right lung and the upper lobe of the left lung. Bronchoscopy revealed thick white mucous plugs in the left lingular bronchus, which could be removed partially by suctioning. The culture of bronchoalveolar lavage fluid and bronchoscopy brush specimens grew cottony white mold in sabouraud dextrose agar. Pneumonia caused by *S. commune* was diagnosed based on clinical features and microbial methods.

**Interventions and outcomes:**

Voriconazole combined with inhaled budesonide and formoterol (inhaled corticosteroids + long-acting β-2 receptor agonist) were given, and his symptoms improved. The count of peripheral blood eosinophils and serum total immunoglobulin E decreased after 1 month. Repeated chest computed tomography showed remarkable improvement over the previous lesions.

**Lessons:**

Although rarely reported, infections in the lungs caused by *S commune* should be reminded especially in patients with immunocompromised. This case illustrates the risk factors, clinical symptoms and imaging features of the pneumonia caused by *S. commune*. It also further highlights the diagnosis and treatment of this disease through reviewing relevant literature.

## 1. Introduction

*Schizophyllum commune (S. commune*) is an occasional pathogen which associated with immunocompromised patients such as diabetes, hematological malignancies, long-term used of immunosuppressant and structure lung diseases.^[1,2]^ Few of cases have been reported worldwide since 1950 and most of cases were the form of case report.^[3]^ Respiratory tract is one of the most vulnerable organs to *S. Commune* infections resulting in sinusitis, allergic bronchopulmonary mycosis (ABPM) and pneumonia which is closely related to continuous inhalation of fungal hyphae. Despite of the increasing number of cases of *S. Commune* infections to pulmoanry recent years, most cases were reported from Japan.^[4–18]^ In addition, there is still unclear about the clinical presentation, imaging, therapeutic agents and prognosis of pneumonia to *S. Commune*. We herein reported a rare case of pulmonary mycosis due to *S. Commune* in a diabetic patient and relevant published global literature were also reviewed. The study was approved by the Institutional Ethics Board of Shaoxing People’s Hospital and the patient has provided informed consent for publication of the case.

## 2. Case report

**Figure 1.**
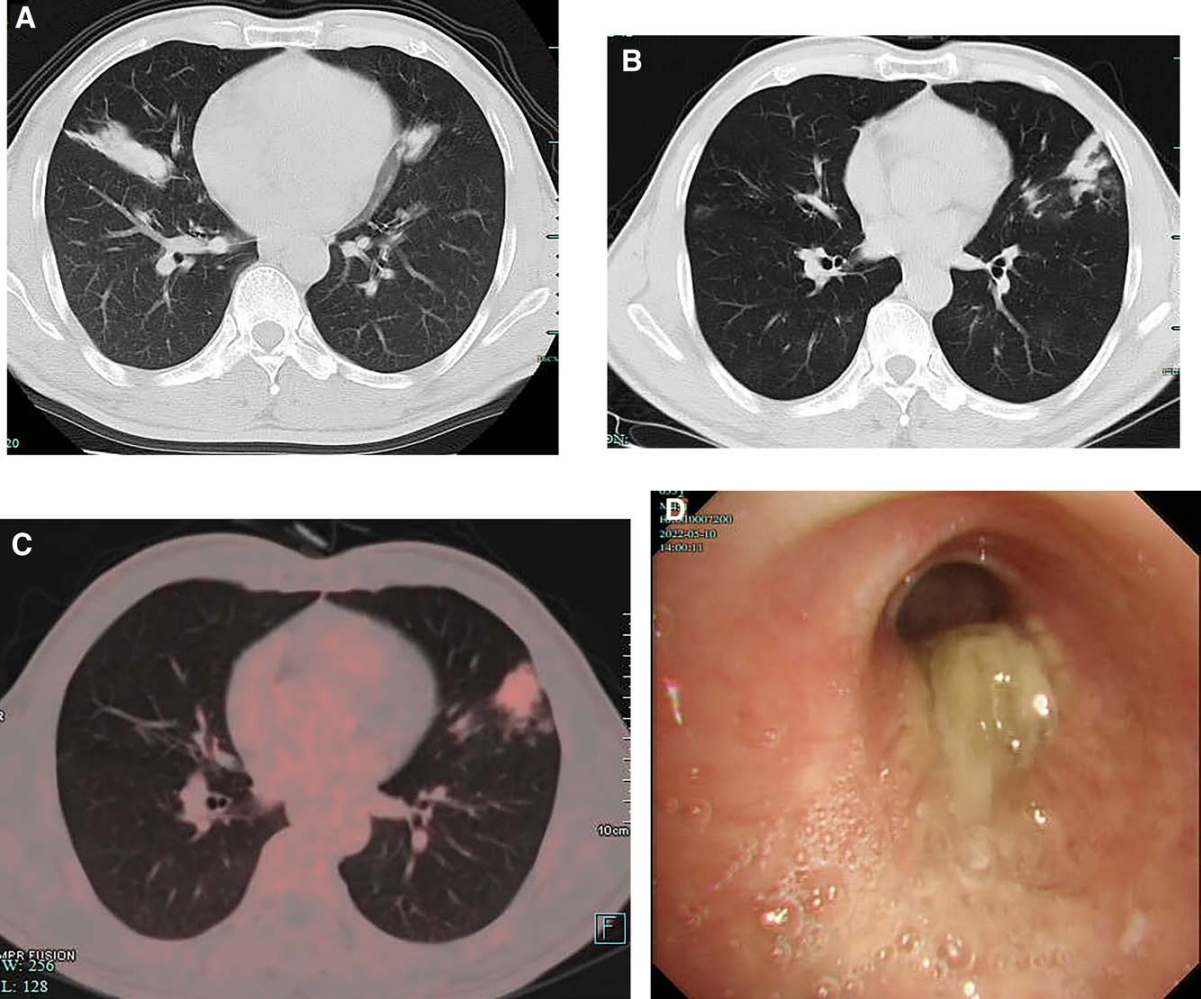
(A) Chest computed tomography (CT) showed gloved finger sign, consolidation in the middle lobe of the right lung and upper lobe of the left lung 6 years ago. (B) A patchy of consolidation, infiltrates and gloved finger sign were seen in the left lingular lobe on Chest CT. (C) PET/CT showed mild metabolic activity with standard uptake value (SUV max 3.5) in the left lingular lobe which consistent with inflammation. (D) Bronchoscopy showed mucosal swelling and thick white mucous plugs in the left lingular bronchus which could be removed partially by suctioning.

A 55-year-old male with a history of diabetes and poor glycemic control presented to our hospital with cough and sputum for half a month. He has been smoking for 50 years. What’s more, he had been come to our outpatient for persistent cough and sputum 6 years ago. Laboratory test showed white blood cell count of 7.7 × 10^9^/L with 8.4% of eosinophils at that time. Chest computed tomography (CT) showed gloved finger sign, consolidation in the middle lobe of the right lung and upper lobe of the left lung (Fig. 1A). He was prescribed with cefuroxime for 14 days and the symptoms improved. Unfortunately, he was lost to follow-up later. On admission, no abnormal signs identified. Laboratory test revealed white blood cell count of 4.02 × 10^9^/L with 18.8% of eosinophils, elevated absolute eosinophil count of 0.76 × 10^9^/L and high total serum immunoglobulin E (IgE) level (327 KU/L). Serum biochemistry, β-D-1,3-glucan, cryptococcus serum antigen, interferon-gamma release assays, C-reactive protein and respiratory pathogens testing was normal. Pulmonary function test indicated neither asthma nor chronic obstructive pulmonary disease. Chest CT disclosed a patchy of consolidation, infiltrates and gloved finger sign in the left lingular lobe (Fig. 1B). PET/CT showed mild metabolic activity in the left lingular lobe which consistent with inflammation indicating the exclusion of malignancy (Fig. 1C). Bronchoscopy showed mucosal swelling and thick white mucous plugs in the left lingular bronchus which could be removed partially by suctioning (Fig. 1D). The eosinophils of bronchoalveolar lavage fluid (BALF) was 10% and culture of BALF was performed. Differential diagnosis of ABPM or allergic bronchopulmonary aspergillosis and other infectious diseases should be considered owing to elevated serum eosinophil count, high total IgE and mucous plugs in bronchus.

**Figure 2.**
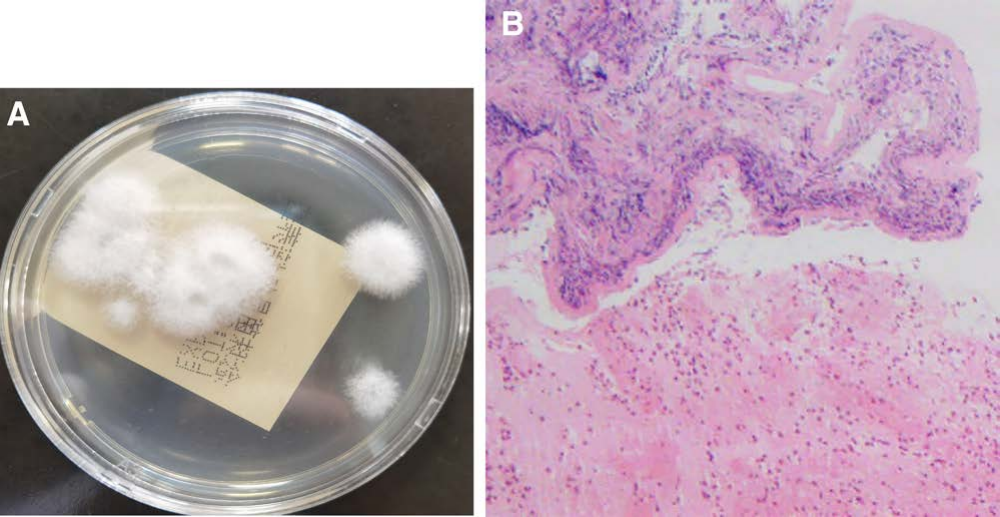
(A) White, woolly colonies of *Schizophyllum commune* in sabouraud dextrose agar after incubation. (B) Large amounts of eosinophils were seen in biopsied tissue.

The culture of BALF and brush demonstrated filamentous, and cottony white mold grew in sabouraud dextrose agar both of BALF and bronchoscopic brush specimens (Fig. 2A). Large amounts of eosinophils were seen in biopsied tissue with hematoxylin and eosin stain (Fig. 2B). Nucleotide sequencing (18S rRNA) identified this fungus as *S. Commune*. Antifungal susceptibility test of the culture of BALF was performed using the broth micro dilution method according to the Clinical and Laboratory Standards Institute. Minimum inhibitory concentrations of antifungal agents was presented in Table 1. He was given voriconazole combined with inhaled corticosteroids/formoterol long-acting β-2 receptor agonist. One month after treatment, his symptoms disappeared and the level of peripheral blood eosinophils, serum total IgE decreased. Repeated chest CT showed remarkable absorption of the previous lesion. He continued the original treatment for 3 months and neither side-effect of drugs nor recurrence occurred.

**Table 1:** In vitro antifungal susceptibility test for *Schizophyllum commune*.

Antifungal agents	MIC (μg/mL)
FLU	16
VOR	0.03
CAS	8
5-FC	0.5
ITR	0.5
AMB	0.25
MFG	8
POS	1

5-FC = 5-Fluorocytosine, AMB = amphotericin B, CAS = caspofungin, FLU = fluconazole, ITR = itraconazole, MFG = micafungin, MIC = minimum inhibitory concentration, POS = posaconazole, VOR = voriconazole.

## 3. Discussion

Aspergillus is one of the most common opportunistic pathogens associated with morbidity and mortality in immunosuppressed patients worldwide.^[19]^ Nonetheless, the incidence of rare mold infections other than aspergillosis have been gradually increased owing to the widespread use of antibiotics and antifungal agents.^[20]^ *Schizophyllum commune (S. commune*) is a fungi of Basidiomycete which commonly existed in rotting wood and leaves.^[1,21]^ Infections due to *S. commune* was first reported by Kligman in 1950.^[22]^ It is an opportunistic pathogen that yield to the patients according to the age, immune status and the load of the pathogens in the host. Respiratory tract (especially lungs) is the major target of *S. commune.*^[12]^ Previous studies^[1,21]^ showed the incidence of infections caused by *S. commune* might be underestimated because of unawareness of this specimen and the difficulties in laboratory identification. Herein, literature search was performed using the databases of “Pubmed,” “Medline” and “Web of science” and using the search strategy: “Pulmonary” or “Lung” combined with “*Schizophyllum commune*” from 1994 to 2022. A total of 23 articles and 25 cases were enrolled (Table 2). In addition, there were several reports in Japanese which were excluded because of not available of the full text.

**Table 2:** Literature review of pneumonia due to *Schizophyllum commune*

		Year	Nation	Sex	Age	History	Symptom	Phisical examination	Lab examination	Image (CT)	Site	Bronchoscopic examination	Treatment	Duration	Prognosis
1	Kamei^[4]^	1994	Japan	F	57	Sinusitis	Productive cough	Neg	WBC: 4,100 mm^3^ with 12% eosinophils; total IgE: 4286U/ml; Sputum analysis: eosinophils and Charcot-Leyden crystals; serum antibody to *S commune*: 1:320	Infiltration, nodule	RUL	Neg	None	None	Recurrence
2	Sigler^[2]^	1995	1995	F	53	TB, DM	Chronic cough, hemoptysis	NR	Neg	Nodule	RUL	Neg	Surgery	None	NR
3	Amitani^[5]^	1996	Japan	F	67	TB	Cough, wheezing	Rhonchi	WBC: 5300/mm^3^ with 12% eosinophils	Gloved finger sign	LUL, LLL	Mucous plugs	Remove mucus plugs by bronchoscope	NR	Improved but reccurence later
4	Miyazaki^[6]^	2000	Japan	F	51	Bronchiectasis	Cough, hemoptysis	Breathing sound diminished	WBC:10,700/mm^3^	Infected bullae	Both upper lobes	Mucous plugs	Amphotericin B	1 mo	Improved and no recurrence
5	Lizasa^[7]^	2001	Japan	M	74	Tuberculosis, hypertension	Routine medical examination	Neg	Positve of IgG antibody to *S commune*	Irregular-shaped consolidation	RUL	Membranous obstruction	Surgery	None	No recurrence
6	Kawayama^[8]^	2003	Japan	F	74	DM, hypertension	Cough, wheezing, dyspnea and fever	Coarse, crackling rales	WBC: 10,310/mm^3^ witn 12% eosinophils; ESR:67 mm/h; CRP: 15.5mg/mL, high percentage (58%) of eosinophils of BALF	Consolidation, infiltrates	Both upper lobes	Oedematous tracheobronchial mucosa	Inhaled corticosteroid	NR	Improved but pathogen still positive from BALF
7	Bulajic^[23]^	2006	UK	M	56	None	Routine medical examination	Neg	WBC: 6.800/mm^3^ witn 1.6% eosinophils	Smooth wall syst	LUL	Neg	Surgery	None	Improved and no recurrence
8	Ishiguro^[9]^	2007	Japan	F	54	None	Cough, sputum	Neg	WBC: 3.400/mm^3^ with 9.5% eosinophils	Mucos plugs and consolidation	Both upper lobes	Thick mucos plugs	Itraconazole + removal of the mucos plugs by bronchoscope	3 mo	Improved and no recurrence
9	Tullio^[24]^	2008	Italy	M	59	Gastric carcinoma, Hodgkin disease	Routine medical examination	Neg	WBC: 19.450/mm^3^	Infiltrates	NR	NR	Fluconazole	6 wk	Improved and no recurrence
10	Roan^[25]^	2009	Taiwan	F	56	Cardiac transplantation	Routine medical examination	Neg	Neg	Multiple solid nodules	Bilateral lungs	NR	Surgery + fluconazole	1 mo	Improved and no recurrence
11	Ogawa^[10]^	2011	Japan	F	71	Asthma	Cough, wheezing and dyspnea	Wheezes and rhonchi	WBC: 8,500/mm^3^ with 0.7% eosinophils	Neg	NR	NR	Itraconazole + theophylline + montelukast sodium + ICS/LABA/LAMA	NR	Improved and no recurrence
12	Ogawa^[11]^	2012	Japan	F	58	None	Cough, sputum	Neg	WBC: 4.900/mm^3^ with 20.9% eosinophils, total IgE: 805U/mL, positive for serum specific IgG and IgE	Mucous plugs	LLL	Thick green mucous plugs	Remove the mucus plugs with bronchoscopy	NR	Improved and no recurrence
13		2012	Japan	F	70	None	Cough, fever	Neg	WBC: 14,500/mm^3^ with 9.4% eosinophils, total IgE: 787U/mL, 77% eosinophils of sputum, positive for serum specific IgG and IgE	Mucous impaction	RUL	Neg	Itraconazole	3 mo	Improved and no recurrence
14	Chowdhary^[1]^	2013	India	F	35	None	Cough, expectoration, breathlessness and wheezing	Coarse crepitations rhonchi	Increased eosinophils, serum total IgE: 2,448U/mL, positive specific IgE to *S commune*	Bilateral dilatation of central bronchi	All lobes	Neg	ICS/LABA	NR	Improved and no recurrence
15		2013	North American	M	42	TB, DM	Haemoptysis	Pallor	4% eosinophils, positive for specific IgE to *S commune*	Air-crescent	RUL	Neg	Itraconazole + deflazocort	NR	Improved and no recurrence
16	Chan^[26]^	2014	Hong Kong	M	78	TB, COPD, CHF, hypertension, gout	Fever, productive cough and dyspnea	Stony dullness on percussion, reduced breath sound	WBC: 21,470/mm^3^, neutrophils: 20.20mm^3^, CRP: 59mg/mL, ESR: 132mm/h	Irregular soft tissue, air-fluid sign with pleural effusion	Right lung	NR	Voriconazole	NR	Died
17	Seki^[12]^	2014	Japan	F	61	None	Cough, breathlessness, wheezing	Coarse crepitations rhonchi	11.9% eosinophils, eosinophil count: 861/mL, positive of specific IgE for Aspergillus spp	Bronchiectasis and mucus plugs	RUL	NR	itraconazole + prednisolone	NR	Improved
18	Kobayashi^[13]^	2016	Japan	F	80	TB	Cough, dyspnea	SpO2: 89% at room air	Eosinophil count: 651/mL	Mucoid impaction	Left lung	Thick mucus plug	Expectorant agent (bromhexine)	20 mo	Improved and no recurrence
19	Shen^[21]^	2016	China	F	59	Nasal polypectomy	Cough, yellow phlegm	NR	WBC: 6.300/μL with 7.6% of eosinophils, serum CEA: 8.1ng/mL, total IgE: 883KU/L	Gloved finger sign, consolidation and bronchial occlusion	RML	Mucous plugs	Voriconazole + inhaled pulmicort	NR	Improved
20	Ishiguro^[14]^	2018	Japan	F	63	None	Cough, sputum	Neg	Total IgE: 1,363IU/mL; positive of Serum IgE and IgG to *S commune*	Mucoid impaction	Left lingual bronchus	Mucoid plug	Itraconazole initially, but changed to voriconazole later	1 yr	Improved and no recurrence
21	Ito^[15]^	2019	Japan	M	42	Asthma	Productive cough	NR	Eosinophil: 1.700/uL, total IgE 69IU/mL, positive of IgG to S.commune	Central bronchiectasis and infiltrates	RUL, RLL, left lingual bronchus, LLL	Mucous plugs	Itraconazole + prednisolone	3 mo	Recurred initially, then combined with prednisolone
22	Ihoh^[16]^	2021	Japan	M	76	DM, hypertension	Hemosputum	Neg	WBC: 7.090/μL, eosinophil: 177/μL	Cavity with internal nodule	LUL	NR	Surgery	None	Improved and no recurrence
23	Zhu^[3]^	2021	Canada	M	56	None	Cough, chest pain and hemoptysis	NR	NR	Air-containing collection with pleural thickening and calcification	Left lower lobe	Copious pus	Surgery + voriconazole	NR	Improved and no recurrence
24	Kim^[17]^	2022	Korea	M	73	AML	Fever	Multiple whitish plaques in oropharyngeal mucos	Eosinophilia and ANC count: 0	Consolidations, GGO	Both lungs	NR	Itraconazole	8 wk	Improved and no recurrence
25	Yamaguchi^[18]^	2022	Japan	F	78	None	cough	NR	Elevation of serum IgE, CEA and eosinophil count	Mucous impaction	RML	Mucus plugs	Corticosteroid	5 mo	Improved and no recurrence; CEA decreased

AML = acute myeloid leukemia, ANC = absolute neutrophil count, BALF = bronchoalveolar lavage fluid, CT = computed tomography, CEA = carcinoembryonic antigen, CHF = congestive heart failure, COPD = chronic obstructive pulmonary disease, DM = diabetes mellitus, ESR = erythrocyte sedimentation rate, F = female, GGO = ground-glass opacities, ICS/LABA = inhaled corticosteroids/long-acting β-agonist, ICS/LABA/LAMA = inhaled corticosteroids/long-acting β-agonist/long-acting muscarine antagonist, IgE = immunoglobulin E, LLL = left lower lobe, LUL = left upper lobe, M = male, Neg = normal, NR = not reported, RLL = right lower lobe, RML = right middle lobe, RUL = right upper lobe, TB = tuberculosis, WBC = white blood cell.

Most of the cases (60%, n = 15) were from Japan suggesting Japan was more concerned about this pathogen. However, few of cases were reported in China. Nearly 2 thirds of the patients (16 of 25) had certain underlying conditions. The patient we present also had a history of diabetes and poor glycemic control which was a high-risk factor of *S. commune* infection. The main clinical symptoms were atypical, including cough, sputum, wheezing, dyspnea and hemoptysis. Four patients were asymptomatic and found in routine medical examination. As to laboratory examination, nearly half (12/25) of the patients showed elevation of eosinophils, 7 of 25 patients showed increased serum total IgE, 10 of 25 patients revealed elevation of serum antibody to *S. commune* (including specific IgE and IgG).In addition, elevated serum aspergillus fumigatus IgG and/or serum aspergillus fumigatus IgE in ABPM due to *S. commune* were found in some cases, which might be related to the presence of antigenic cross-reactivity between *S. commune* and aspergillus fumigatus.^[11,14]^

The imaging of chest CT of pneumonia caused by *S. commune* can be classified into followings according to the literature: Mucous plugs or glove finger sign. These were the most common manifestation; Pulmonary fungal balls or uniformly dense strips. That might be the result of the growth of *S. commune* on the basis of the exsisting pulmonary cavity; Pulmonary atelectasis, caused by complete blockage of bronchus by mucous plugs; Consolidations or infiltrates; Pleural effusion. While someone with normal chest CT mainly presented with airway hyperresponsiveness.^[10]^ As to our case, chest CT showed gloved finger sign and consolidation in the bilateral lungs 6 years ago. Combined with clinical symptoms, high eosinophils and CT image, infection of *S. commune* should be suspected at that time, despite no further examination performed. The typical manifestation of bronchoscopy was jelly-like substances or mucus plug in the bronchial lumen which could not be removed by forceps or suction causing obstruct the bronchus and/or local pulmonary atelectasis in several cases.^[9,15,18,21]^

The diagnosis of pneumonia of *S. commune* was mainly based on the culture of respiratory or postoperative lung specimens.^[3]^ While negative culture results have also been reported and serum antibody to *S commune* is helpful.^[11]^ Recently, accurate identification has been increasingly achieved by molecular methods using nucleotide sequencing of the internal transcribed spacer, 18S rRNA gene and/or large subunit rRNA gene.^[1,26]^ While metagenomic next-generation sequencing is gradually applied in the detection of *S. commune*.^[12]^ It can be misdiagnosed as invasive pulmonary aspergillosis or allergic bronchopulmonary aspergillosis/ABPM because of the similar clinical manifestations and imaging.^[4]^ In our case, pathogen of *S. commune* was cultured in both sputum and BALF, although elevated serum eosinophil count and high total IgE and mucous plugs, the diagnostic criteria of ABPM was not met.^[27]^

The optimal antifungal therapy and the course of treatment have been limited. Both itraconazole and voriconazole were successfully used, while a part of cases achieved good results with fluconazole and amphotericin B. It had reported that voriconazole had good curative effect on *S. commune* infection and could be used as a rescue therapy for ineffective of itraconazole. ^[12,14]^ In addition, there is currently no guideline recommendation on antifungal agents in combination with systemic or inhale corticosteroid. Based on previous literature, if one had symptoms such as shortness of breath, wheezing, dyspnea, elevation of blood eosinophils and serum total IgE, combined systemic or inhale corticosteroid was recommended but not alone. The duration of the treatment for *S. commune* infection is not standardized ranging from 1 month to 1 year, and usually 3 to 4 months suggesting individualized treatment course was considered. The prognosis of pulmonary of *S commune* infection is well. Only 1 ptatient in literature review died which may be associated with her advanced age, underlying diseases and a wide range of lesions involved.^[26]^

## 4. Conclusion

In conclusion, clinicians should pay great attention to pulmonary infections caused by *S. commune* especially in immunocompromised patients despite few cases have been reported. Azole antifungals are the preferred agents for *S. commune* infection. Good prognosis will be achieved under proper antifungal therapy and the course of the treatment should be individualized.

## Author contributions

**Conceptualization:** Xing Chen.

**Data curation:** Xing Chen, YeFeng Chen.

**Formal analysis:** Jian Sun, YeFeng Chen.

**Investigation:** YeFeng Chen.

**Methodology:** Jie Wang.

**Project administration:** ShuYing Liu.

**Supervision:** Jie Wang.

**Writing – original draft:** Xing Chen.

**Writing – review & editing:** Jian Sun, YeFeng Chen, Jie Wang, ShuYing Liu.
